# Literacy shapes thought: the case of event representation in different cultures

**DOI:** 10.3389/fpsyg.2014.00290

**Published:** 2014-04-16

**Authors:** Christian Dobel, Stefanie Enriquez-Geppert, Pienie Zwitserlood, Jens Bölte

**Affiliations:** ^1^Institute for Biomagnetism and Biosignalanalysis, Westfälische Wilhelms-Universität MünsterMünster, Germany; ^2^Department of Experimental Psychology, European Medical School, Carl von Ossietzky UniversityOldenburg, Germany; ^3^Institute for Psychology, Westfälische Wilhelms-Universität MünsterMünster, Germany

**Keywords:** action representation, conceptual processes, Yucatec Maya

## Abstract

There has been a lively debate whether conceptual representations of actions or scenes follow a left-to-right spatial transient when participants depict such events or scenes. It was even suggested that conceptualizing the agent on the left side represents a universal. We review the current literature with an emphasis on event representation and on cross-cultural studies. While there is quite some evidence for spatial bias for representations of events and scenes in diverse cultures, their extent and direction depend on task demands, one‘s native language, and importantly, on reading and writing direction. Whether transients arise only in subject-verb-object languages, due to their linear sentential position of event participants, is still an open issue. We investigated a group of illiterate speakers of Yucatec Maya, a language with a predominant verb-object-subject structure. They were compared to illiterate native speakers of Spanish. Neither group displayed a spatial transient. Given the current literature, we argue that learning to read and write has a strong impact on representations of actions and scenes. Thus, while it is still under debate whether language shapes thought, there is firm evidence that literacy does.

The influence of language on perception and thought is one of the most fascinating topics in the cognitive sciences and troubles researchers at least since Sapir and Whorf (Whorf, 1956). In their view, termed linguistic relativity, learning and knowing a language has a profound impact on cognitive domains such as basic perception (for recent reviews see Regier and Kay, 2009; Lupyan, 2012), recognition memory (e.g., Fausey and Boroditsky, 2011) or spatial processing (e.g., Levinson, 2003a,b). These results did not remain undisputed and were objected by other influential researchers (e.g., Pinker, 1994; Li and Gleitman, 2002). Some argue that language follows universal principles and that semantics are based on an innate “language of thought” (Fodor, 1975). Thus, the relation of language and thought is still hotly debated (e.g., Gentner and Goldin-Meadow, 2003), with the general favor swinging back and forth in one or the other direction.

One way to tackle this controversy is to investigate whether representations of actions or static scenes follow a spatial direction and if such bias depends on language, that is, on the (syntactically constrained) order of mention of scene protagonists in the sentence. The idea that action representations have a spatial direction was inspired by an aphasic patient, who assigned the agent role to the left figure in action events, independent whether this figure was the agent or the patient in the action. This finding was corroborated by data from a sentence-to-picture matching task, where his performance was best when the agent was depicted on the left, and the action-transient went from left to right (Maher et al., 1993; Chatterjee et al., 1995). In follow-up studies, this finding generalized to native speakers of English, who displayed a similar left-to-right action transient when depicting action events (Chatterjee et al., 1995, 1999). Thus, with respect to the tight connection between language and space, this agent-left preference might constitute a universal characteristic, present in the vast majority of the world's languages (Chatterjee, 2001) and maybe even influencing artistic works (Chatterjee, 2002) or calling fouls in soccer (Kranjec et al., 2010). Such a left-to-right order was not only found for action events involving animate agents, but also in scenes consisting of arrangements of inanimate objects (Jahn et al., 2007). Jahn and colleagues proposed that listeners build a mental model of first-mentioned objects (e.g., “A table is between the TV and a chair”) and then try to spatially integrate the next-mentioned objects (e.g., “The light is on the left of the TV”). Their data suggest that first-mentioned objects are represented in a left-to-right fashion in the preferred initial model. The authors also assumed that this spatial bias appears as a consequence of the habitual reading and writing direction (RWD). There is already a lot of evidence that RWD has an impact on a rather large number of cognitive processes involving both low-level skills and higher-level representations and processes. Examples are perceptual span (Pollatsek et al., 1981), scanning direction (Chokron and Imbert, 1993), representations of numbers (Dehaene et al., 1993), time (Tversky et al., 1991) and aesthetic preferences (Chokron and De Agostini, 2000; for a more comprehensive list see Román et al., 2013). Presenting a review about directionality effects in various domains and tasks, Vaid (2010) concludes that a motoric account which is influenced by RWD is the most parsimonious explanation.

Obviously, possible confounds of RWD have to be taken into account when investigating the agent-left bias. With tasks very similar to Chatterjee et al. (1995, 1999), Maass and Russo (2003) compared Italian and Arab speakers. Experience of Arab speakers ranged from exclusive exposure to right-to-left RWD (Arabic), to studying in a culture with left-to-right RWD (Italy). The results clearly demonstrated that RWD heavily influences the tendency to conceptualize agents on the left or right of scenes. Italians located them on the left, while Arabs with exclusive right-to-left RWD placed them on the right. The amount of experience with a particular RWD modulated this tendency in an almost linear fashion. Nevertheless, the left-to-right tendency was more strongly expressed in Italians than the right-to-left tendency in Arabs with exclusive experience of this RWD. Thus, spatial predispositions for agents might be innate, but they are strongly modulated by cultural factors. Very similar results were found for Spanish and Arab speakers who had to interpret and draw descriptions of static scenes (Román et al., 2013).

However, the tendency to place agents on the left in cultures with left-to-right RWD and on the right in right-to-left RWD cultures was not reported in all studies. Altmann et al. (2006) presented English and Arabic speakers with action verbs in either active or passive voice (e.g., “chase” or “is chased”), and asked them to subsequently draw stick figures illustrating the action. In contrast to earlier studies, participants from both cultures neither displayed an effect of RWD nor of active/passive voice. English speakers drew the agent more often on the right side when sketching passive verbs, but no other biases were found.

How can this contradictory finding be accounted for? One reason may be the format of linguistic input: single verbs or verbs embedded in sentences. In an earlier study, we only found a spatial transient for verbs embedded in sentence context (Dobel et al., 2011). Thus, to observe a spatial bias, the linguistic input must have the format of a sentence or, alternatively, an ordered list of objects. Altmann et al. (2006) surmised that the lack of a spatial bias was due to their participants interpreting the verbs more statically than dynamically. This seems unlikely given the literature on static scenes, where spatial preferences were reported (e.g., Román et al., 2013). Furthermore, action verbs by themselves are dynamic in nature, as they always imply a change of state (for an overview see Dobel et al., 2010).

To our knowledge, the only other study that reported no consistent spatial bias investigated Korean participants that had either learned to read vertically from right-to-left, or horizontally from left-to-right (Barrett et al., 2002). These participants also drew images in response to sentences describing action events. An explanation for the null finding for participants with vertical right-to-left RWD might be that they were considerably older (> 60 years of age) than the control group. In fact, they were older than all other groups reported so far. There is indeed evidence that spatial biases, as measured with line-bisection tasks, disappear in older participants (Jewell and McCourt, 2000). Another explanation might be that the vertical reading direction conflicted with the horizontal drawing direction, and thus failed to induce a consistent transient. Barrett et al. (2002) assumed that the horizontal left-to-right RWD group was exposed to more than one scanning direction when learning to read, and that this failed to evoke a spatial bias. This explanation is supported by studies on participants subjected to variable amounts of experience with a specific RWD (Maass and Russo, 2003; Román et al., 2013). Both studies found that mixed experience with different RWDs leads to weaker spatial preferences.

From the perspective of innateness, one might predict a spatial bias for placing agents left to be present in preschoolers, who have far less exposure to a prominent RWD than adults. We addressed this question and found opposing biases in adult speakers of German and Hebrew, as already predicted by earlier studies. However, these spatial preferences were absent in illiterate preschoolers (Dobel et al., 2007). We also manipulated the order in which agents and patients (for transitives) or agents and recipients (for ditransitives) were presented by comparing active and passive voice. This manipulation did not influence Hebrew speakers, but German preschoolers and adults were affected by order-of-mention: first-mentioned event participants were more likely to be placed left.

Furthermore, although Hebrew is written from right to left, it might not be the best language to investigate directionality effects. Letters are written from left-to-right as is arithmetic and musical notation. As such a comparison of speakers of languages such as Urdu (right-to-left) and Hindi (left-to-right) might evidence even stronger directional biases than a comparison of speakers of Hebrew and some Germanic language (Vaid, 1995).

Using a different approach, we obtained further evidence against a universal predisposition with speakers of German Sign Language (DGS), who actually use space to convey meaning (Dobel et al., 2011). In signed language, a spatial preference might be more readily visible than in spoken language. As in the studies above, participants read event descriptions, and illustrated their interpretation either by drawing or arranging toy figures. We tested two types of verbs that differ in the way they are signed. Verbs with a horizontal transient are typically signed in a left-to-right manner, seen from the addressee's point of view (e.g., as in “giving” where the agent is typically signed to the right of the signer and the patient to the left). Verbs with sagittal transients, in contrast, are signed moving away from, or toward the speaker (e.g., as in “pushing,” where the direction of movement iconically represents onset and end points of a physical motion, assigning semantic, and syntactic roles to these points). For horizontal transients, signers placed agents at the same position in space as in the signed message. They thus showed a direct mapping preference for verbs with horizontal transients, by putting agents at the same position in space as in the signed message. This effect was not present for verbs following a sagittal transient. Taken together, spatial preferences in building conceptual-semantic representations are modulated by cultural and language-related factors, as well as by short-lived situational factors such as grammar.

Although these studies seem to deliver a clear-cut message, we wish to point out some caveats. First, as stressed by Román et al. (2013), all studies so far investigated speakers of languages with a dominant subject-verb-object (SVO) word order. We demonstrated that word order biases the spatial representation of actions (Dobel et al., 2007). A simple first-in-first-out strategy and representing events as they appeared follows pragmatic principles (Grice, 1975) and thus leads to a spatial arrangement of event participants. Second, the claim that learning to read and write induces spatial transients for action representations needs support from illiterate adults, not only from preschoolers. Even if consistent action transients are innate, they might reach their full manifestation only in adulthood and/or only after experience with reading and writing sets the necessary parameter for a spatial transient. To fill some of these gaps, we investigated monolingual speakers of Yucatec Maya, a language spoken by about 800.000–1.2 million people in the Yucatán peninsula, a south-eastern state in Mexico (Lewis, 2009). The most important characteristic for our purposes is that Yucatec Maya has a predominant verb-object-subject (VOS) word order (e.g., England, 1991; Bolles and Bolles, 2004). The passive voice is a common structure in this language (Verhoeven, 2007).

Twelve Yucatec speakers (mean age = 57 years; 4 men) were compared to 12 monolingual speakers of Spanish (mean age = 57; 3 men), from the state of Veracruz (Eastern part of Mexico). All participants were right handed (Oldfield, 1971). Importantly, members of both language groups received no formal schooling and were illiterate. As in earlier studies, participants were asked to draw or to arrange toy figures in response to spoken descriptions of action events. Sentences were read in active or passive voice, and described actions toward or away from an agent, with eight sentences in each condition (see Examples). There were 64 trials for each participant consisting of 16 different actions (comprising transitive and ditransitive verbs) with the *transient* of movement either away or toward the agent (e.g., away: to kick, to push; toward: to pull, to take). Each sentence was presented in active and passive voice and the content of each sentence had to be drawn or little toy figures had to be arranged. Actors were “woman,” “man,” “boy” and “girl.” The order of sentences was balanced such that maximally two items from the same condition occurred in succession. A separate list of trials was created for each participant. Testing was performed individually at the participant's home in presence of two experimenters, one being a native speaker of Yucatec. Informing participants about the study, getting their approval and testing lasted about 60 min.

Examples (agent marked in italics, verb in bold, recipient/patient underlined):

1. Direction of movement from agent to recipient, active voice.

Spanish: Un *niño* **regala** flores a una niña

Yucatec: **Ku síij lool ti**‘jun túul chan x ch‘úupal jun túul *chan xi‘ipal*.

English: *A boy* **gives** flowers to a girl.

2. Direction of movement from agent to recipient, passive voice.

Spanish sentence: Un niño es **empujado** por *una mujer*.

Yucatec: **Ku túulch'inta'al** jun túul chan xi'ipal tumeen jun túul *ko'olel*.

English: A boy is **pushed** by *a woman*.

3. Direction of movement from patient to agent, active voice.

Spanish: *Una mujer* **deja** a una niña.

Yucatec: **Ku p'atik** jun túul chan x ch'úupal jun túul *ko'olel*.

English: *A woman* **leaves** a girl.

4. Direction of movement from patient to agent, passive voice.

Spanish: Un niño es **jalado** por *un hombre*.

Yucatec: **Ku kóola'al** jun túul chan xi‘ipal tumeen jun túul *wíinik*.

English: A boy is **pulled** by *a man*.

Based on earlier results, we expected at least for the speakers of Spanish an influence of the factors *transient* (more agent-left placements if the action transient moves away from the agent; Chatterjee, 2001), *voice* (more agent-left placements in active voice; Dobel et al., 2007), and *task* (more agent-left placements in drawing than in arranging; cf. Dobel et al., 2011). If there is indeed an innate left-to-right predisposition, we should find evidence for this in speakers of Yucatec as well, possibly similarly mediated by transient, voice, and task. In contrast, if a spatial bias depends on learning to read and write, there should be no evidence for such a representational bias in either group.

The results from the repeated measurement ANOVAs (based on arcsine transformed ratios of agent-left placements) with the above-mentioned factors, however, showed only a significant effect of task [*F*_(1, 22)_ = 9.095; *p* = 0.006]. More agent-left placements were observed in drawing than in arranging (a similar effect was reported in Dobel et al., 2011). None of the other main effects and interactions became significant. Taking a statistically very liberal approach, comparing each condition in each language against chance (see Figure 1 for an illustration of results), revealed that only one comparison reached significance (drawing of actions away from agent in active voice for Yucatec speakers [*t*_(11)_ = 2.6; *p* = 0.025; *r* = 0.61; 63% agent-left placements]. In the seven studies investigating the bias for agents using similar tasks as here (Chatterjee et al., 1995; Barrett et al., 2002; Maass and Russo, 2003; Altmann et al., 2006; Dobel et al., 2007, 2011; Román et al., 2013), ten out of eleven comparisons were significant. In light of this, the effect seems to be rather strong and easy to find if present. Thus, even though the literature on event representation in adults made strong predictions about the presence of an agent-left bias, at least in speakers of Spanish, these hypotheses were not supported.

**Figure 1:**
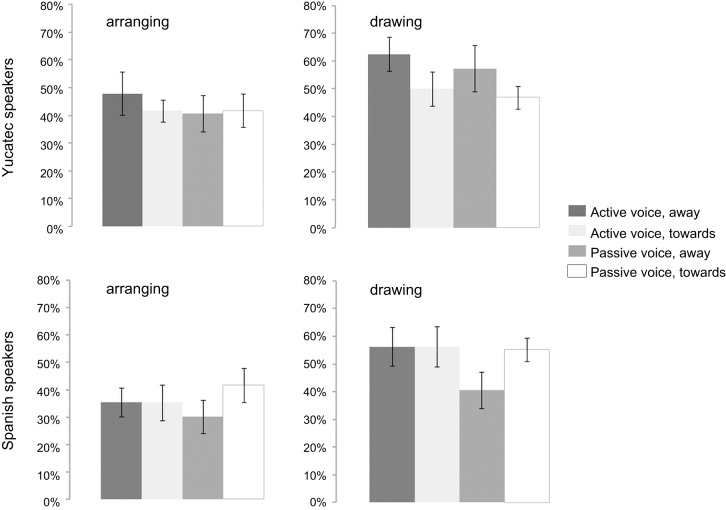
**Percentage left-placements as a function of condition and language (*t*-bars represent one SE)**.

In conclusion, quite a few studies argued for spatial preferences in event and scene representation. Their extent depend on one's native language, short-lived contextual and situational factors (such as task, or word order, based on language-specific syntactic factors) and, most importantly, reading and writing direction. It was still an open question how these factors would be mediated by a language using VOS structure. Here, we found no evidence for a spatial preference in persons speaking such a language. We also observed no bias in illiterate speakers of Spanish, an SVO language. Thus, a mere first-in first-out strategy imposed by SVO languages, as argued above, is not sufficient to induce a spatial transient of action representation. As literacy distinguishes our groups from those of earlier studies, we conclude that a spatial bias in action representations is brought forth only if one learns to read and write. Note that there was also no spatial bias in preschoolers who had not yet learned to read and write (Dobel et al., 2007). If literacy happens, it has a very strong impact on a wide range of mental processes, some of which were listed above. In recent years, these finding from the cognitive sciences were supported by neuroscientific evidence demonstrating that learning to read and write has a profound impact on brain function (e.g., Dehaene et al., 2010) and even on the neural architecture (Carreiras et al., 2009). Thus, while it is still under debate whether language shapes thought (for opposing views see e.g., Pinker, 1994; Levinson, 2003a,b), there is now firm evidence that literacy does. Given the recent interest in non-verbal cognitive processes in bilinguals (e.g., De Groot, 2010; Cook and Bassetti, 2011), we consider the investigation of bilingual speakers that possess corresponding reading and writing skills with opposing directions as highly interesting. If the spatial bias is as context and task dependent as was shown in several studies, then the language in which the stimulus material is presented will exert an effect that is the product of the RWD associated with that language. Such a finding would provide strong evidence that RWD mediates between language and thought even if reading and writing is not at play.

## Conflict of interest statement

The authors declare that the research was conducted in the absence of any commercial or financial relationships that could be construed as a potential conflict of interest.
